# Global trends and disparities in the burden of heart failure caused by chronic kidney disease: an analysis of the global burden of disease study 2021

**DOI:** 10.3389/fmed.2025.1567128

**Published:** 2025-04-17

**Authors:** Lingxia Ye, Xin Huang, Yufeng Xu

**Affiliations:** ^1^Department of Endocrinology and Metabolism, The Second Affiliated Hospital of Zhejiang University School of Medicine, Hangzhou, Zhejiang, China; ^2^Department of Endocrinology and Metabolism, The Fifth Affiliated Hospital of Wenzhou Medical University, Lishui Municipal Central Hospital, Lishui, China; ^3^Department of Ophthalmology, The Second Affiliated Hospital of Zhejiang University School of Medicine, Hangzhou, Zhejiang, China

**Keywords:** heart failure, chronic kidney disease, burden of disease, years lived with disability, disparity

## Abstract

**Background:**

Heart failure (HF) is a major public health concern, and chronic kidney disease (CKD) plays a significant role in its pathogenesis. Understanding the trends and disparities in the burden of HF caused by CKD can provide valuable insights into health policymaking.

**Methods:**

This study was a secondary analysis based on previously published data. We obtained global, regional, national, and age- and sex-specific data on the prevalence and years lived with disability (YLDs) of HF caused by CKD from the Global Burden of Disease Study 2021 (GBD 2021) and performed a secondary comparative analysis by age, sex, time, location, sociodemographic index (SDI), and health system level.

**Results:**

In 2021, there were 1,936.9 (95%UI: 1,600.2–2,343.5) thousand cases of HF caused by CKD globally, with an age-standardized rate of YLDs of 3.1 (95%UI: 1.9–4.4) per 100,000 population. The global burden of HF caused by CKD has continuously increased from 1990 to 2021 and is expected to keep growing through 2045 according to predictions. Significant disparities were found across different locations, genders, and ages. Higher burdens were noted among males, older individuals, and regions with lower SDI or less advanced health systems.

**Conclusion:**

The burden of HF caused by CKD has increased significantly since 1990 and varies widely across regions. More significant efforts are needed in the prevention and treatment of CKD and HF, especially among older individuals and males in regions with lower SDI or less advanced health systems.

## Background

Heart failure (HF) is recognized as a global public epidemic. It is a complex syndrome resulting from a myriad of diseases that affect the heart and is one of the leading causes of hospital admissions worldwide (1). Given the significant impact of HF, substantial progress has been made in recent decades in understanding its epidemiology, pathophysiology, diagnosis, and treatment, with ongoing rapid advancements. According to a recent study (2), there were approximately 56.19 million prevalent cases of HF globally in 2019, with an age-standardized rate of 711.9 per 100,000 population, resulting in 5.05 million years lived with disability (YLDs) and an age-standardized rate of 63.92 per 100,000 population. A wide range of cardiac conditions, hereditary defects, and systemic diseases can result in HF, and patients with HF can have mixed etiologies, which are not mutually exclusive.

Chronic kidney disease (CKD) is a progressive condition characterized by a reduction in kidney function due to various causes and is a significant contributor to non-communicable diseases. A recent study (3) showed that there were approximately 18.99 million CKD cases globally in 2019, with the age-standardized incidence rate reaching 233.65 per 100,000 population and the age-standardized incidence rate of disability-adjusted life years (DALYs) reaching 514.86 per 100,000 population. CKD is a leading cause of HF. A recent systematic meta-analysis, which included over 1 million individuals, demonstrated that the estimated glomerular filtration rate (eGFR) and urinary albumin-to-creatinine ratio (ACR) were both closely associated with the risk of all-cause and CV death (4, 5). The increased cardiovascular risk in CKD is multifactorial, including hypertension, diabetes, renal anemia, increased vascular stiffness, and low-grade inflammation (5, 6). As Gansevoort et al. (6) suggested, the strong causal association between chronic kidney disease and cardiovascular risk implies that preventing the progression of chronic kidney disease is, by definition, preventing cardiovascular disease.

Assessing the disease burden of heart failure can inform public health policymaking and improve healthcare delivery systems. However, to the best of our knowledge, no systematic studies have analyzed the burden of HF caused by CKD at the global, regional, and national levels. To fill this gap, the present study, using the Global Burden of Disease (GBD) approach, aims to provide a systematic analysis of the global, regional, and national burden of HF caused by CKD from 1990 to 2021, with projections through 2045.

## Methods

### Study design and data sources

This study is a secondary analysis based on previously published data (7). It is based on publicly available data and does not contain any personally identifying information; thus, ethical approval was not required. Data on the prevalence and YLD of heart failure were obtained from the GBD 2021 study.^1^ The GBD study provided detailed epidemiological estimates for causes of death, disability, and associated risk factors, delineated by age, sex, year, and geographical location, and was updated annually.

### Case definition

In the GBD study, heart failure was recognized as an impairment and diagnosed using criteria such as the Framingham criteria or European Society of Cardiology criteria (7). According to the severity of the disease, HF was categorized into four levels: treated HF, mild HF, moderate HF, and severe HF. Moreover, 27 underlying causes of HF were identified, including ischemic heart disease, hypertensive heart disease, chronic obstructive pulmonary disease (COPD), rheumatic heart disease, congenital heart abnormalities, atrial fibrillation, and chronic kidney disease (7).

YLDs were calculated using a microsimulation process that used estimated age-sex-location-year-specific prevalent counts of non-fatal disease sequelae (consequences of a disease or injury) for each cause, along with disability weights for each sequela as inputs (7). The sociodemographic index (SDI) was applied as a composite indicator of social and economic conditions. It is the geometric mean of 0–1 indices, including the fertility rate among females younger than 25 years, the average years of education for those aged 15 years or older, and lagged distributed income per capita (7), with values ranging from 0 to 1. The quintiles include low SDI (<0.45), low-middle SDI (≥0.45 and <0.60), middle SDI (≥0.60 and <0.69), high-middle SDI (≥0.69 and <0.805), and high SDI (≥0.80).

### Statistical analysis

The prevalence and YLD estimates were extracted in absolute numbers, rates, and percentages, stratified by region, country, territory, age, and sex. The data are presented as values with a 95% uncertainty interval (UI). The age-standardized rate (ASR) of YLDs was expressed as the number per 100,000 population. The Kruskal–Wallis test was used with non-normal distributions to evaluate the difference in age-standardized rates between males and females. The autoregressive integrated moving average (ARIMA) model, widely used in time series analysis (8, 9), was applied to estimate the burden of heart failure caused by chronic kidney disease from 2021 to 2045 (R system, version 4.2.2; detailed method in Supplementary File 1). All statistical analyses, except those specified above, were conducted using Prism software version 9.0 (GraphPad, San Diego, CA, USA). A *p*-value less than 0.05 was considered statistically significant.

## Results

### Global burden of HF caused by CKD from 1990 to 2021

Globally, in 2019, the prevalence of HF caused by CKD was 617.8 (95%UI: 505.1–746.5) thousand, with an ASR of 13.6 (95%UI: 11.2–16.2) per 100,000 population. In 2021, the prevalence number increased to 1,936.9 (95%UI: 1,600.2–2,343.5) thousand, with an ASR of 24.2 (95%UI: 19.9–29.2) per 100,000 population, representing an average annual change rate of 2.52% compared to 1990. The number of YLDs of HF caused by CKD in 1990 was 79.0 (95%UI: 50.9–115.0) thousand, with an ASR of 1.7 (95%UI: 1.1–2.6) per 100,000 population. By 2021, the YLDs number rose to 245.7 (95%UI: 154.6–356.7) thousand, with an ASR of 3.1 (95%UI: 1.9–4.4) per 100,000 population, reflecting an average annual growth rate of 2.5%.

**Figure 1:**
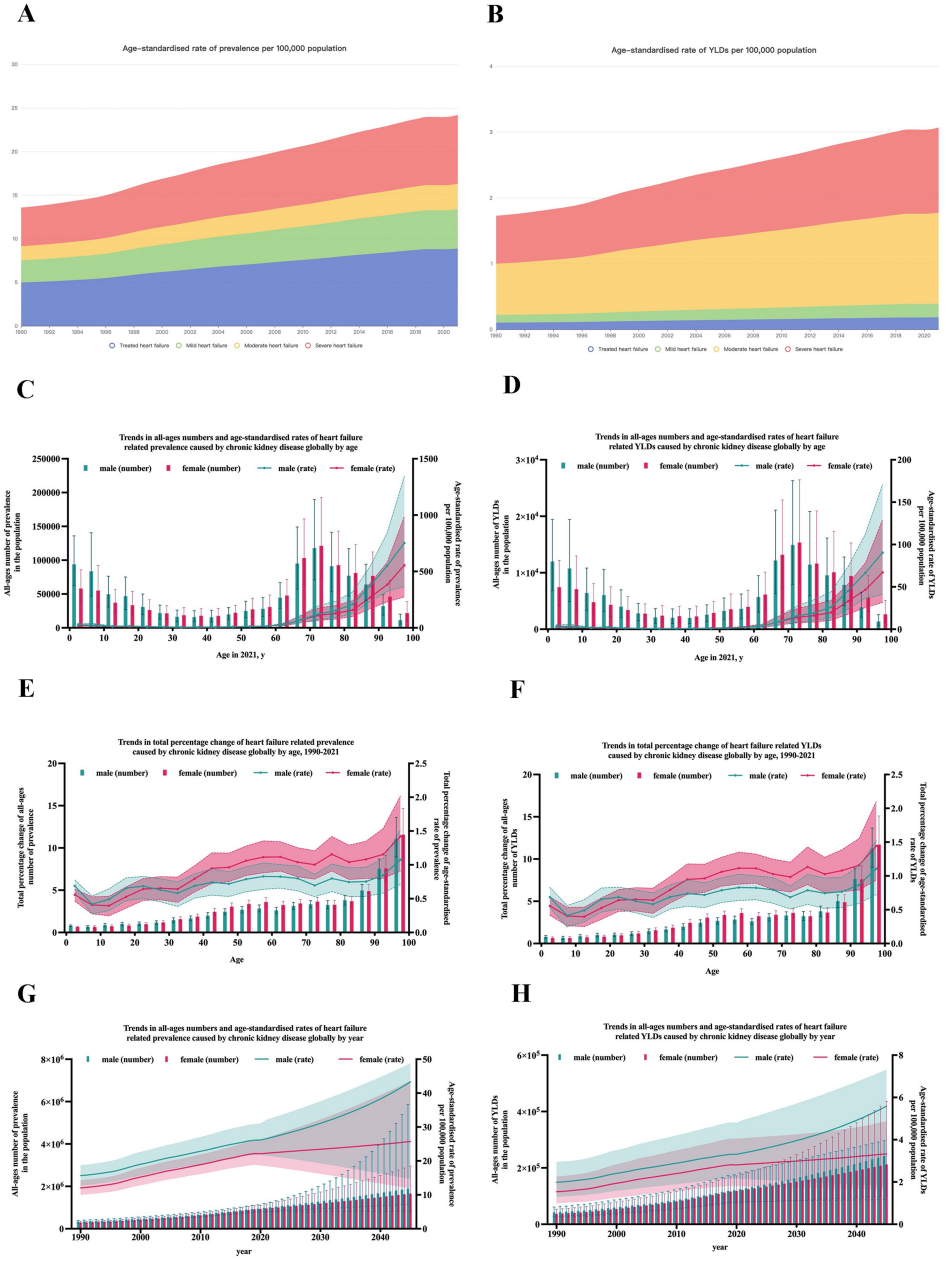
Prevalence and YLDs of heart failure caused by chronic kidney disease globally. **(A)** ASR of the prevalence of HF caused by CKD by severity from 1990 to 2021; **(B)** ASR of YLDs of HF caused by CKD by severity from 1990 to 2021; **(C)** trends in the prevalence of HF caused by CKD by gender and age; **(D)** trends in YLDs of HF caused by CKD by gender and age; **(E)** trends in total percentage change of prevalence of HF caused by CKD by gender and age; **(F)** trends in total percentage change of YLDs of HF caused by CKD by gender and age; **(G)** trends and prediction in the prevalence of HF caused by CKD by gender and year; **(H)** trends and prediction in YLDs of HF caused by CKD by gender and year. YLDs, years lived with disability; ASR, age-standardized rate; HF, heart failure; CKD, chronic kidney disease.

In the GBD study, the severity of heart failure was divided into four levels. In 1990, the prevalence number of HF included treated HF [227.0 (95%UI: 183.7–273.1) thousand], mild HF [115.2 (95%UI: 81.6–158.6) thousand], moderate HF [74.5 (95%UI: 52.0–101.0) thousand], and severe HF [201.2 (95%UI: 159.6–247.2) thousand] (Table 1 and Supplementary Table 1 in Supplementary File 2). Moreover, the ASR of prevalence per 100,000 population was disparate at different severity levels: treated HF [5.0 (95%UI: 4.0–6.0)], mild HF [2.5 (95%UI: 1.8–3.4)], moderate HF [1.6 (95%UI: 1.2–2.2)], and severe HF [4.4 (95%UI: 3.5–5.4)]. In 2021, the ASR of prevalence per 100,000 population of treated HF [8.9 (95%UI: 7.2–10.8)], mild HF [4.5 (95%UI: 3.2–6.1)], moderate HF [2.9 (95%UI: 2.1–4.0)], and severe HF [24.2 (95%UI: 19.9–29.2)], all increased dramatically compared to 1990. Moreover, the ASR (per 100,000 population) of YLDs in 2021 [treated HF: 1.3 (95% UI: 0.80–1.90); mild HF: 0.2 (95% UI: 0.1–0.3); moderate HF: 0.2 (95% UI: 0.1–0.3); severe HF: 3.1 (95% UI: 1.9–4.4)] varied by severity levels (Figures 1A–B and Supplementary Table 2 in Supplementary File 2). However, there was little difference in the percentage change in the age-standardized prevalence rate from 1990 to 2021 across varying severities [treated HF:2.50%; mild HF: 2.51%; moderate HF: 2.51%; severe HF: 2.5%].

**Table 1:** Burden of heart failure caused by chronic kidney disease in 2021 by regions.

GBD 2021 super regions	Heart failure		Treated heart failure		Mild heart failure		Moderate heart failure		Severe heart failure	
	All-ages number	Age-standardized rate	All-ages number	Age-standardized rate	All-ages number	Age-standardized rate	All-ages number	Age-standardized rate	All-ages number	Age-standardized rate
	(×1,000, 95%UI)	(/100,000, 95% UI)	(×1,000, 95%UI)	(/100,000, 95% UI)	(×1,000, 95%UI)	(/100,000, 95% UI)	(×1,000, 95%UI)	(/100,000, 95% UI)	(×1,000, 95%UI)	(/100,000, 95% UI)
**Global**	245.70 (154.57, 356.66)	3.10 (1.90, 4.40)	103.40 (64.69, 149.53)	1.30 (0.80, 1.90)	14.80 (8.27, 23.99)	0.20 (0.10, 0.30)	16.60 (9.70, 26.94)	0.20 (0.10, 0.30)	110.90 (66.85, 171.23)	3.10 (1.90, 4.40)
**Sex**										
Male	124.40 (78.96, 181.89)	3.40 (2.10, 4.90)	52.30 (32.59, 76.24)	1.40 (0.90, 2.00)	7.50 (4.11, 12.10)	0.20 (0.10, 0.30)	8.40 (4.93, 13.75)	0.20 (0.10, 0.40)	56.20 (34.16, 87.12)	3.40 (2.10, 4.90)
Female	121.30 (76.60, 176.76)	2.80 (1.80, 4.10)	51.00 (31.85, 73.11)	1.20 (0.70, 1.70)	7.30 (4.08, 11.86)	0.20 (0.10, 0.30)	8.20 (4.76, 13.19)	0.20 (0.10, 0.30)	54.70 (33.16, 83.52)	2.80 (1.80, 4.10)
**SDI grouping levels**										
High SDI	54.30 (33.92, 81.38)	2.80 (1.80, 4.20)	22.80 (14.24, 33.75)	1.20 (0.70, 1.70)	3.30 (1.71, 5.30)	0.20 (0.10, 0.30)	3.70 (2.09, 6.04)	0.20 (0.10, 0.30)	24.50 (14.20, 38.09)	2.80 (1.80, 4.20)
High-middle SDI	31.20 (19.64, 46.64)	1.90 (1.20, 2.80)	13.10 (8.16, 19.08)	0.80 (0.50, 1.20)	1.90 (1.02, 3.01)	0.10 (0.10, 0.20)	2.10 (1.18, 3.51)	0.10 (0.10, 0.20)	14.10 (8.42, 21.67)	1.90 (1.20, 2.80)
Middle SDI	77.90 (48.71, 112.92)	3.20 (2.00, 4.70)	32.80 (20.77, 47.23)	1.40 (0.90, 2.00)	4.70 (2.57, 7.56)	0.20 (0.10, 0.30)	5.30 (3.04, 8.58)	0.20 (0.10, 0.40)	35.20 (21.38, 52.56)	3.20 (2.00, 4.70)
Low-middle SDI	45.40 (28.94, 65.61)	2.90 (1.80, 4.20)	19.10 (11.98, 27.95)	1.20 (0.80, 1.80)	2.70 (1.50, 4.42)	0.20 (0.10, 0.30)	3.10 (1.75, 4.96)	0.20 (0.10, 0.30)	20.50 (12.42, 30.73)	2.90 (1.80, 4.20)
Low SDI	36.70 (22.15, 54.09)	4.80 (3.00, 7.20)	15.40 (9.34, 22.91)	2.00 (1.30, 3.00)	2.20 (1.18, 3.66)	0.30 (0.20, 0.50)	2.50 (1.41, 4.13)	0.30 (0.20, 0.50)	16.60 (9.60, 25.46)	4.80 (3.00, 7.20)
**Health system grouping levels**										
Advanced Health System	64.70 (40.75, 96.00)	2.50 (1.60, 3.80)	27.20 (17.22, 40.21)	1.10 (0.70, 1.60)	3.90 (2.06, 6.28)	0.20 (0.10, 0.20)	4.40 (2.50, 7.17)	0.20 (0.10, 0.30)	29.20 (17.10, 44.81)	2.50 (1.60, 3.80)
Basic Health System	96.90 (61.22, 140.91)	2.90 (1.90, 4.30)	40.80 (25.69, 58.72)	1.20 (0.80, 1.80)	5.80 (3.19, 9.46)	0.20 (0.10, 0.30)	6.50 (3.72, 10.73)	0.20 (0.10, 0.30)	43.70 (26.71, 65.21)	2.90 (1.90, 4.30)
Limited Health System	72.80 (45.56, 105.34)	3.20 (2.00, 4.70)	30.60 (18.96, 44.66)	1.30 (0.80, 1.90)	4.40 (2.39, 7.22)	0.20 (0.10, 0.30)	4.90 (2.82, 8.00)	0.20 (0.10, 0.30)	32.90 (19.49, 49.57)	3.20 (2.00, 4.70)
Minimal Health System	11.10 (6.56, 16.75)	5.40 (3.20, 8.20)	4.70 (2.76, 7.24)	2.30 (1.40, 3.40)	0.70 (0.35, 1.11)	0.30 (0.20, 0.50)	0.70 (0.41, 1.30)	0.40 (0.20, 0.60)	5.00 (2.93, 7.72)	5.40 (3.20, 8.20)
**High income**										
High-income Asia Pacific	10.40 (6.43, 15.87)	2.30 (1.50, 3.30)	4.40 (2.64, 6.60)	1.00 (0.60, 1.40)	0.60 (0.33, 1.07)	0.10 (0.10, 0.20)	0.70 (0.39, 1.19)	0.20 (0.10, 0.30)	4.70 (2.69, 7.26)	2.30 (1.50, 3.30)
Western Europe	19.90 (12.74, 29.97)	2.20 (1.40, 3.40)	8.40 (5.24, 12.53)	0.90 (0.60, 1.40)	1.20 (0.62, 1.97)	0.10 (0.10, 0.20)	1.30 (0.78, 2.17)	0.20 (0.10, 0.20)	9.00 (5.36, 13.77)	2.20 (1.40, 3.40)
Australasia	1.30 (0.82, 2.00)	2.60 (1.60, 3.80)	0.60 (0.35, 0.85)	1.10 (0.70, 1.60)	0.10 (0.04, 0.14)	0.20 (0.10, 0.30)	0.10 (0.05, 0.15)	0.20 (0.10, 0.30)	0.60 (0.35, 0.93)	2.60 (1.60, 3.80)
High-income North America	20.50 (12.46, 32.13)	3.30 (2.00, 5.10)	8.60 (5.26, 13.54)	1.40 (0.90, 2.10)	1.20 (0.65, 2.08)	0.20 (0.10, 0.30)	1.40 (0.78, 2.37)	0.20 (0.10, 0.40)	9.20 (5.34, 14.50)	3.30 (2.00, 5.10)
Southern Latin America	2.30 (1.39, 3.47)	2.80 (1.70, 4.20)	1.00 (0.58, 1.48)	1.20 (0.70, 1.80)	0.10 (0.07, 0.23)	0.20 (0.10, 0.30)	0.20 (0.09, 0.25)	0.20 (0.10, 0.30)	1.00 (0.61, 1.61)	2.80 (1.70, 4.20)
**Central Europe, Eastern Europe and Central Asia**										
Central Europe	2.30 (1.45, 3.55)	1.60 (1.00, 2.40)	1.00 (0.62, 1.47)	0.70 (0.40, 1.00)	0.10 (0.08, 0.23)	0.10 (0.10, 0.20)	0.20 (0.09, 0.26)	0.10 (0.10, 0.20)	1.10 (0.62, 1.67)	1.60 (1.00, 2.40)
Eastern Europe	1.70 (1.03, 2.65)	0.80 (0.50, 1.20)	0.70 (0.43, 1.08)	0.30 (0.20, 0.50)	0.10 (0.05, 0.17)	0.00 (0.00, 0.10)	0.10 (0.07, 0.20)	0.10 (0.00, 0.10)	0.80 (0.45, 1.25)	0.80 (0.50, 1.20)
Central Asia	1.30 (0.84, 1.84)	1.50 (1.00, 2.10)	0.50 (0.34, 0.80)	0.60 (0.40, 0.90)	0.10 (0.04, 0.13)	0.10 (0.00, 0.10)	0.10 (0.05, 0.14)	0.10 (0.10, 0.20)	0.60 (0.37, 0.90)	1.50 (1.00, 2.10)
**Latin America and the Caribbean**										
Tropical Latin America	6.70 (4.16, 10.22)	2.80 (1.80, 4.30)	2.80 (1.71, 4.19)	1.20 (0.70, 1.80)	0.40 (0.22, 0.64)	0.20 (0.10, 0.30)	0.50 (0.26, 0.77)	0.20 (0.10, 0.30)	3.00 (1.79, 4.78)	2.80 (1.80, 4.30)
Central Latin America	17.30 (10.92, 25.01)	7.00 (4.40, 10.30)	7.30 (4.57, 10.48)	2.90 (1.80, 4.20)	1.00 (0.56, 1.66)	0.40 (0.20, 0.70)	1.20 (0.68, 1.90)	0.50 (0.30, 0.80)	7.80 (4.80, 11.62)	7.00 (4.40, 10.30)
Andean Latin America	4.70 (2.94, 6.67)	7.90 (4.90, 11.20)	2.00 (1.22, 2.85)	3.30 (2.00, 4.80)	0.30 (0.15, 0.46)	0.50 (0.30, 0.80)	0.30 (0.18, 0.50)	0.50 (0.30, 0.90)	2.10 (1.32, 3.21)	7.90 (4.90, 11.20)
Caribbean	1.90 (1.21, 2.78)	3.70 (2.40, 5.50)	0.80 (0.50, 1.17)	1.60 (1.00, 2.30)	0.10 (0.06, 0.19)	0.20 (0.10, 0.40)	0.10 (0.08, 0.20)	0.30 (0.10, 0.40)	0.90 (0.52, 1.33)	3.70 (2.40, 5.50)
**Southeast Asia, East Asia, and Oceania**										
East Asia	34.40 (20.99, 51.77)	1.80 (1.10, 2.70)	14.40 (8.77, 22.01)	0.80 (0.50, 1.10)	2.10 (1.08, 3.40)	0.10 (0.10, 0.20)	2.30 (1.30, 3.93)	0.10 (0.10, 0.20)	15.50 (9.03, 24.26)	1.80 (1.10, 2.70)
Southeast Asia	22.20 (14.22, 31.46)	3.70 (2.40, 5.30)	9.30 (5.84, 13.43)	1.60 (1.00, 2.20)	1.30 (0.73, 2.18)	0.20 (0.10, 0.40)	1.50 (0.87, 2.42)	0.30 (0.10, 0.40)	10.00 (6.24, 14.83)	3.70 (2.40, 5.30)
Oceania	0.10 (0.07, 0.17)	1.40 (0.90, 2.10)	0.00 (0.03, 0.07)	0.60 (0.40, 0.90)	0.00 (0.00, 0.01)	0.10 (0.00, 0.10)	0.00 (0.00, 0.01)	0.10 (0.10, 0.20)	0.10 (0.03, 0.08)	1.40 (0.90, 2.10)
**North Africa and the Middle East**										
North Africa and the Middle East	17.50 (11.39, 24.69)	3.60 (2.30, 5.10)	7.30 (4.75, 10.51)	1.50 (1.00, 2.20)	1.10 (0.59, 1.71)	0.20 (0.10, 0.30)	1.20 (0.68, 1.84)	0.20 (0.10, 0.40)	7.90 (5.08, 11.69)	3.60 (2.30, 5.10)
**South Asia**										
South Asia	25.00 (15.59, 36.37)	1.60 (1.00, 2.40)	10.50 (6.48, 15.48)	0.70 (0.40, 1.00)	1.50 (0.83, 2.45)	0.10 (0.10, 0.20)	1.70 (0.96, 2.74)	0.10 (0.10, 0.20)	11.30 (6.92, 17.55)	1.60 (1.00, 2.40)
**Sub-Saharan Africa**										
Southern Sub-Saharan Africa	3.40 (2.05, 5.18)	5.80 (3.50, 8.90)	1.40 (0.87, 2.12)	2.40 (1.50, 3.70)	0.20 (0.11, 0.34)	0.40 (0.20, 0.60)	0.20 (0.13, 0.39)	0.40 (0.20, 0.70)	1.50 (0.90, 2.40)	5.80 (3.50, 8.90)
Eastern Sub-Saharan Africa	15.60 (9.07, 23.39)	6.10 (3.70, 9.30)	6.60 (3.89, 9.87)	2.60 (1.50, 3.90)	0.90 (0.49, 1.61)	0.40 (0.20, 0.60)	1.10 (0.58, 1.78)	0.40 (0.20, 0.70)	7.00 (3.96, 10.89)	6.10 (3.70, 9.30)
Central Sub-Saharan Africa	5.30 (3.12, 8.10)	6.10 (3.80, 9.30)	2.20 (1.31, 3.48)	2.60 (1.50, 3.90)	0.30 (0.16, 0.53)	0.40 (0.20, 0.60)	0.40 (0.20, 0.60)	0.40 (0.20, 0.70)	2.40 (1.36, 3.72)	6.10 (3.80, 9.30)
Western Sub-Saharan Africa	31.90 (19.40, 46.22)	11.20 (7.00, 16.80)	13.40 (8.09, 19.72)	4.70 (2.90, 6.90)	1.90 (1.01, 3.15)	0.70 (0.40, 1.10)	2.10 (1.25, 3.50)	0.80 (0.40, 1.20)	14.40 (8.47, 21.33)	11.20 (7.00, 16.80)

After classifying CKD according to its causes, the subgroups included CKD due to type 2 diabetes mellitus, type 1 diabetes mellitus, glomerulonephritis, hypertension, and other unspecified causes. As shown in Supplementary Figure 1, in both 1990 and 2021, CKD due to other unspecified causes accounted for the highest burden (58.46% in 1990 and 57.22% in 2021). This was followed by CKD due to type 2 diabetes mellitus (17.10% in 1990 and 17.98% in 2021), CKD due to hypertension (13.48% in 1990 and 14.36% in 2021), and CKD due to glomerulonephritis (10.20% in 1990 and 9.55% in 2021). CKD due to type 1 diabetes mellitus contributed the least burden (0.76% in 1990 and 0.89% in 2021).

### Burden of HF caused by CKD by country and territory

In 2021, the top three countries with the largest prevalence numbers were China [250.0 (95%UI: 192.6–334.9) thousand], Nigeria [180.7 (95%UI: 141.9–219.8) thousand], and the United States [145.2 (95%UI: 106.3–197.7) thousand]. The same countries also had the highest YLD numbers: China [31.9 (95%UI: 19.4–48.4) thousand], Nigeria [22.8 (95%UI: 13.8–32.8) thousand], and the United States [18.3 (95%UI: 11.1–28.7) thousand].

**Figure 2:**
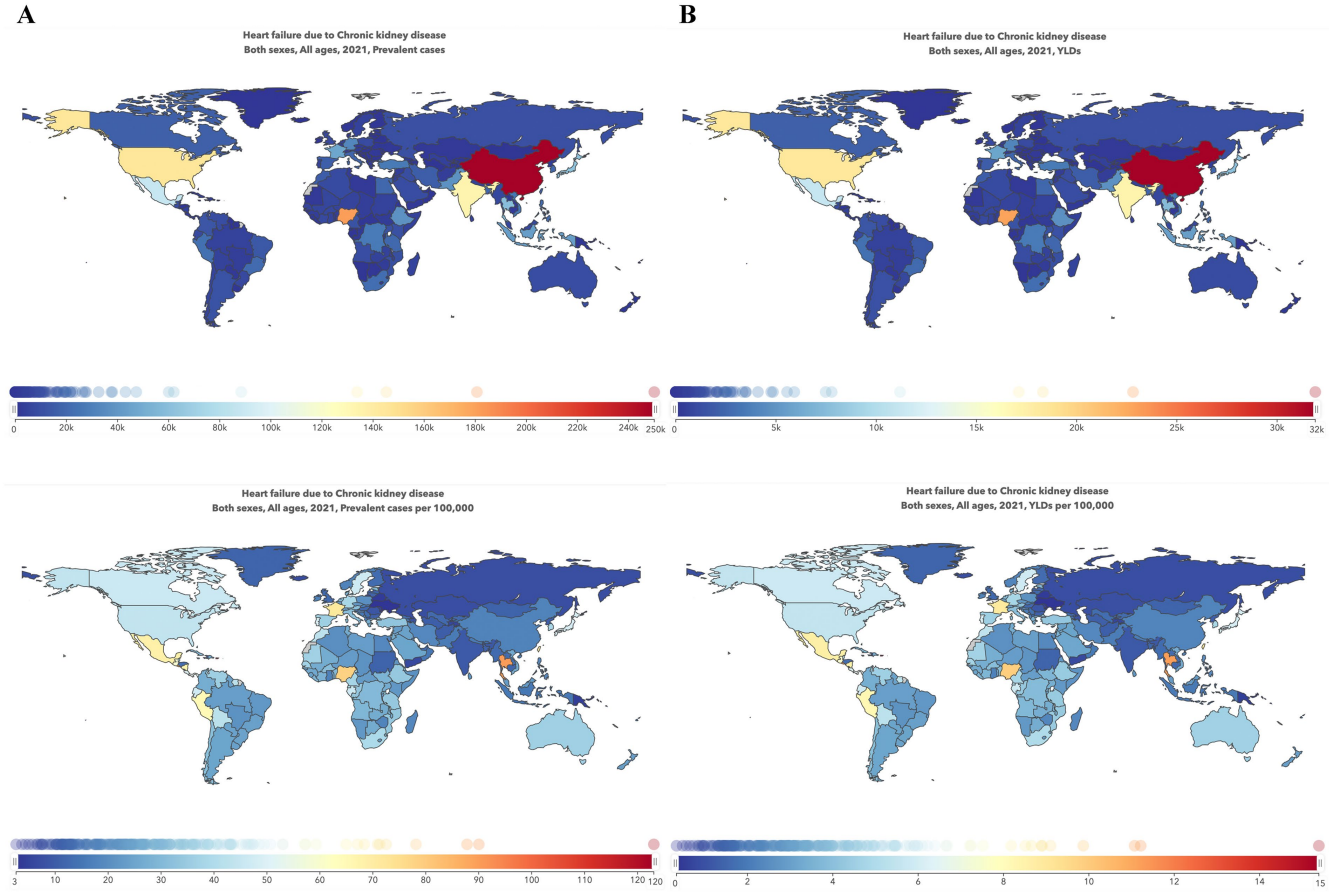
Global maps of the burden of heart failure caused by chronic kidney disease in 2021. **(A)** Prevalence cases and the ASR of HF caused by CKD in 2021; **(B)** YLDs number and ASR of HF caused by CKD in 2021. ASR, age-standardized rate; HF, heart failure; CKD, chronic kidney disease; YLDs, years lived with disability.

After age standardization, the top three countries with the highest ASR of prevalence per 100,000 population were Puerto Rico [123.1 (95%UI: 93.5–158.7)], Thailand [90.0 (95%UI: 70.4–114.1)], and El Salvador [87.8 (95%UI: 72.3–105.8)]. The highest ASR of YLDs was also observed in Puerto Rico [15.4 (95%UI: 9.7–23.2)], Thailand [11.2 (95%UI: 6.9–16.1)], and El Salvador [11.1 (95%UI: 7.0–16.0)], all located near the equator, as shown in Figure 2.

### Burden of HF caused by CKD by SDI

**Figure 3:**
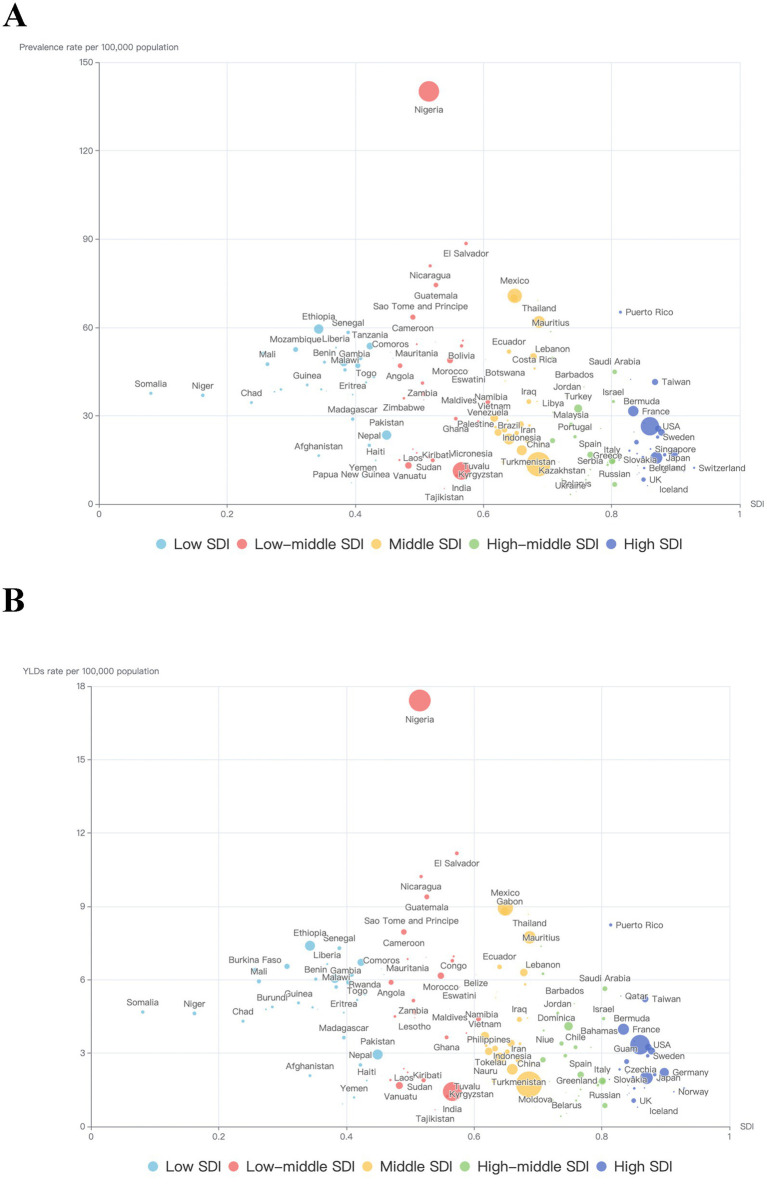
Trends in the burden of heart failure caused by chronic kidney disease by SDI and country in 2021. **(A)** Trends in ASR of prevalence by SDI and country in 2021; **(B)** Trends in the ASR of YLDs by SDI and country in 2021. Each point represents a country or territory, and the size of each point represents the number of prevalence or YLDs. ASR, age-standardized rate; SDI, Sociodemographic Index; YLDs, Years lived with disability.

Figure 3 shows the burden of HF by countries or territories, classified by SDI. As this scattergram shows, there appears to be a trend of higher burden for those with middle SDI or lower SDI. To further investigate the possible connection between socioeconomic status and the burden of HF, Supplementary Figure 2 shows the association between SDI and YLDs of HF caused by CKD by region from 1990 to 2021. Overall, the relationship between SDI and the burden of HF caused by CKD was L-shaped, with the lowest burden observed in the high-middle SDI group (SDI 0.7–0.8) and the highest burden observed in the low SDI group (SDI lower than 0.3).

### Burden of HF caused by CKD at the health system level

**Figure 4:**
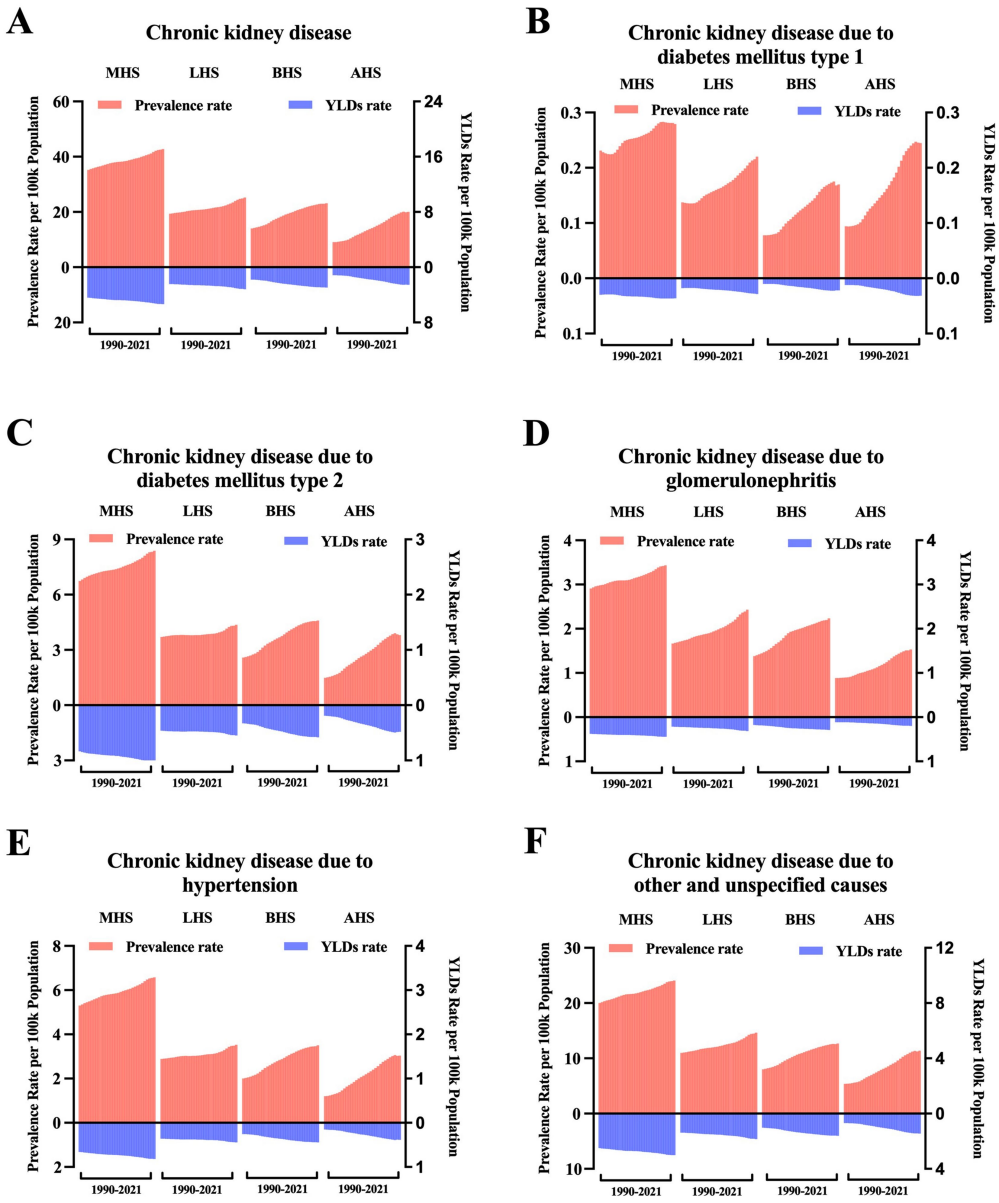
Trends in the age-standardized rate of the burden of heart failure caused by chronic kidney disease at the health system grouping level. **(A)** ASR of prevalence and YLDs of HF caused by CKD by health system grouping level; **(B)** ASR of prevalence and YLDs of HF caused by CKD due to type 1 diabetes mellitus by health system grouping level; **(C)** ASR of prevalence and YLDs of HF caused by CKD due to type 2 diabetes mellitus by health system grouping level; **(D)** ASR of prevalence and YLDs of HF caused by CKD due to glomerulonephritis by health system grouping level; **(E)** ASR of prevalence and YLDs of HF caused by CKD due to hypertension by health system grouping level; and **(F)** ASR of prevalence and YLDs of HF caused by CKD due to other and unspecified causes by health system grouping level. ASR, age-standardized rate; YLDs, years lived with disability; MHS, minimal health system; LHS, limited health system; BHS, basic health system; AHS, advanced health system.

The GBD study 2021 provided information on health system levels by location. As shown in Figure 4A, the overall HF burden caused by CKD is ranked by time in each health system level subgroup. Moreover, from 1990 to 2021, regions with minimal health systems were accompanied by the highest burden, and regions with advanced health systems were accompanied by the lowest burden. After being classified according to the causes of CKD, subgroups including CKD due to type 2 diabetes mellitus, glomerulonephritis, hypertension, and other unspecified causes (Figures 4C–F) demonstrate consistency with the overall trend, except for CKD due to type 1 diabetes mellitus. In this subgroup, regions with minimal health systems experienced the highest burden as well, followed by regions with advanced health systems, and regions with basic health systems presented the lowest burden (Figure 4B).

### Global burden of HF caused by CKD by age and sex

For age, both the global numbers and the age-standardized rates of prevalence and YLDs of HF caused by CKD show significant inequality at different age levels. The highest prevalence of YLDs was observed in individuals aged 70–75 years, with another peak in those aged under 10 years (Figures 1C–D). The lowest burden was observed in individuals aged 30–40 years. After 75 years of age, both the prevalence and YLD rates gradually declined with age. To assess overall growth with time, percentage changes from 1990 to 2021 were calculated. As shown in Figures 1E–F, the percentage change in burden increased dramatically with age.

For gender inequality, the burden of HF, both prevalence and YLDs, caused by CKD in females and males increased with time from 1990 to 2021 (Figures 1G–H). Males consistently had a higher burden than females, particularly after the age of 60 years (Figures 1C–D).

### Future prediction of the global burden of HF caused by CKD

By 2045, the number of people with HF caused by CKD is predicted to increase to 8.25 million (95% UI: 7.22–11.14). As shown in Figures 1G–H and Supplementary Table 3 in Supplementary File 2, by 2045, it is estimated that there will be approximately 1,662.2 (95% UI: 820.2–2,956.6) thousand female cases and 1,892.7 (95% UI: 1,136.6–5,865.8) thousand male cases. The ASR of prevalence is expected to reach approximately 25.7 (95% UI: 11.9–43.6) in females and 43.3 (95% UI: 14.6–48.8) per 100,000 population in males. The ASR of YLDs is predicted to reach approximately 3.3 (95% UI: 1.6–4.9) in females and 5.6 (95% UI: 1.8–7.3) in males.

## Discussion

The GBD study provides the most comprehensive compilation and analysis of global health information available. This study thoroughly evaluated the global burden of HF caused by CKD from 1990 to 2021 and compared different regions, locations, sex, age, SDI, and health system levels based on the GBD data.

Overall, from 1990 to 2021, the burden of HF caused by CKD, measured in prevalence and YLDs, increased substantially. In 2021, globally, there were approximately 1,936.9 thousand prevalence cases and 245.7 YLD cases, with ASRs of prevalence and YLDs reaching 24.2 and 3.1 per 100,000 population, respectively. This indicates that HF caused by CKD is emerging as a pandemic with a significant health burden. According to the predictions, the burden will continue to increase over the next 20 years. Different from a previous study that showed a decreasing trend in the overall ASR of HF (2), our study showed an increasing trend in the burden of HF caused by CKD, indicating that even with much progress made in the management of HF, there is still a need for much more effort in the management of CKD.

Our study revealed significant epidemiological heterogeneity among countries and territories. Geographically, the highest burden was observed in countries near the equator. This may be attributed to several environmental factors, such as climate change, exposure to heavy metals, fluoride, and toxins in food and water, which contribute to chronic non-communicable diseases, including cancer and CKD (10). Moreover, the unstable and short-term geomagnetic field could also be a hazard to cardiac health (11). Additionally, these regions are often characterized by poor economies, further exacerbating the health burden.

In terms of socioeconomic status and health system levels, the highest burden was observed in countries with low SDI and minimal health systems. This is consistent with previous studies showing higher burdens of CKD and HF in low SDI regions (12, 13). Notably, when CKD was categorized by its underlying causes, in contrast to other causes, the burden of HF caused by CKD due to type 1 diabetes mellitus was higher in high SDI countries. Similarly, Gong et al. (14) also noticed that countries with higher SDI exhibited higher type 1 diabetes mellitus prevalence rates in 2019, demonstrating that ethnic differences and migration between countries may play a role in this trend. This may be partly because, although recognized as a disease triggered by autoimmune, genetic risk factors also contribute to its onset (15). Therefore, more emphasis should be given to countries with lower SDI or those located near the equator.

Our study detected significant gender disparities, with males consistently bearing a higher burden than females, especially after the age of 55 years. This gender disparity in the overall burden of HF varies slightly across various studies (2, 16). Previous studies identified that females had a higher prevalence of non-ischemic etiology (which in turn conferred improved survival), higher left ventricular EF, and a lower occurrence of atrial fibrillation (17, 18), which conferred a better prognosis.

HF and CKD were both age-related diseases, with peaks observed in individuals aged 70–75 years and under 10 years. Higher burdens of HF and CKD in the elderly population were also observed by Bragazzi et al. (13) and Xie et al. (12) The peak age under 10 years is primarily due to pediatric HF, which results from a variety of congenital and acquired diseases, including congenital structural dysfunction, acquired inflammation diseases, and prior exposure to chemotherapeutic agents (19).

HF and CKD have a complex interplay, with heart failure often complicating renal disease. The two conditions influence each other, a phenomenon known as cardiorenal syndrome (CRS) (20). As data showed, more than 60% of patients admitted for acute decompensated heart failure suffer from CKD (21), and CKD, in turn, is one of the most significant risk factors for mortality in patients with acute decompensated heart failure (22, 23). Although the strong association between CKD and specific CVD outcomes is not fully understood, a series of mechanisms may play a role (24). Classical studies of the underlying mechanisms of CRS have mainly focused on shared risk factors such as diabetes and hypertension (25), hemostatic abnormalities, anemia (26–28), sodium and volume overload (29, 30), metabolic nutritional changes such as mineral and bone metabolism abnormalities (31), and the presence of uremic toxins (32–35). Recent studies highlighted novel molecular mechanisms of CRS, such as inflammation and activated oxidative stress (36, 37), TGF-β1/Smad signaling pathway (38, 39), Wnt/β-catenin signaling pathway (40, 41), hyperactive renin–angiotensin–aldosterone system (RAAS) (42, 43), dysbiosis of the gut microbiota (44, 45), and dysregulation of non-coding RNAs (46, 47). Due to complex pathophysiological mechanisms, the treatment of CRS is full of challenges. Recent studies have focused on identifying predictors of outcomes and found that higher serum magnesium concentrations are associated with lower risks of death from fatal HF, CHD, and stroke in non-dialysis patients with CKD stages 4 and 5 (48). Additionally, inflammation-based scores, especially the Prognostic Nutritional Index, may be a useful clinical biomarker for CKD progression in CKD with CHF patients (49). In addition, in patients with advanced CKD, the treatment of heart failure requires special attention because many randomized controlled trials of heart failure treatment have not included patients with advanced kidney disease. However, there are still many gaps in both the understanding and treatment of CRS.

The findings of this study have significant implications for clinical practice and public health policy. The increasing burden of HF caused by CKD underscores the need for more comprehensive prevention and treatment strategies, particularly in regions with lower SDI and minimal health systems. Public health programs should focus on early detection and management of CKD to mitigate its impact on HF. Additionally, efforts should be directed toward improving healthcare infrastructure and access in regions with the highest burden. Future research should focus on identifying specific risk factors and interventions that can effectively reduce the burden of HF caused by CKD. Studies should also explore the role of emerging therapies and technologies in improving outcomes for patients with CRS.

This study systematically analyzed the burden of HF caused by CKD at the global, regional, and national levels. However, several limitations should be noted. First, this study assessed epidemiologic trends globally, regionally, and nationally, which may overlook micro-level trends, especially in countries with large populations. Second, this study relied on the data from the GBD study, while the data estimates in the GBD study are derived from various data collection methods and sources. In some less-developed countries, data collection may be absent or sparse, which could lead to heterogeneity and affect the data quality in this study. Third, various HF phenotypes, such as HF with preserved or reduced ejection fraction, which have been the focus of many studies, were not specifically listed, as HF was categorized into four levels according to the severity in the GBD study.

## Conclusion

In conclusion, this study reveals the global health burden of HF caused by CKD from 1990 to 2021, highlighting significant disparities across regions, sex, age, SDIs, and health system levels. The burden is expected to continue increasing until 2045, emphasizing the need for more focused efforts in the prevention and treatment of CKD and HF. We hope this study will inform policymaking and guide future research and clinical practice.

## Data Availability

The original contributions presented in the study are included in the article/Supplementary material, further inquiries can be directed to the corresponding authors.

## Footnotes

^1^ https://vizhub.healthdata.org/gbd-results/

## Supplementary material

SUPPLEMENTARY FIGURE 1 Proportion of heart failure YLDs attributable to chronic kidney disease by various causes. YLDs, Years lived with disability; YLDs are shown as age-standardized rate.

SUPPLEMENTARY FIGURE 2 Trends in YLDs of heart failure caused by chronic kidney disease for 21 GBD regions by SDI, 1990–2021. For each region, points from left to right depict estimates from each year from 1990 to 2021. ASR, age-standardized rate; SDI, Socio-demographic Index; YLDs, Years lived with disability.
