# Supplementary figures and images for: Global trends and disparities in the burden of heart failure caused by chronic kidney disease: an analysis of the global burden of disease study 2021
Source: Front Med (Lausanne). 2025 Apr 17;12:1567128. doi: 10.3389/fmed.2025.1567128 (PMC12043581; doi:10.3389/fmed.2025.1567128)

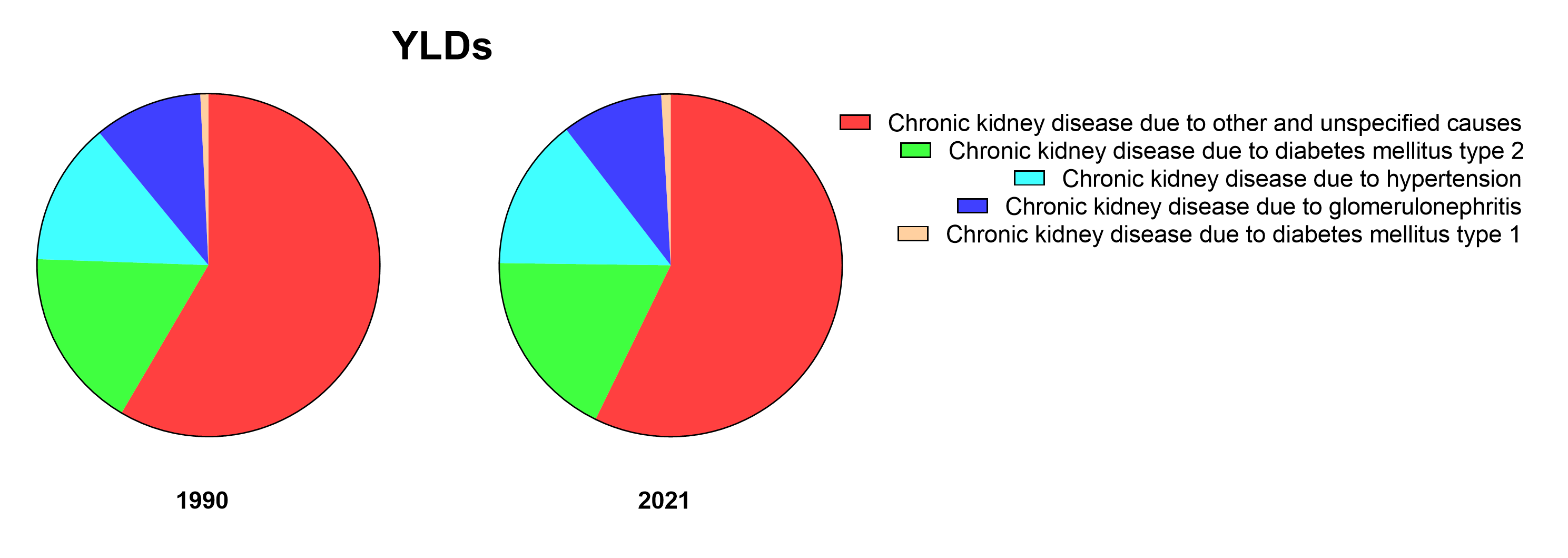

Supplement: SUPPLEMENTARY FIGURE 1 — Proportion of heart failure YLDs attributable to chronic kidney disease by various causes. YLDs, Years lived with disability; YLDs are shown as age-standardized rate. [file Image_1.tif]

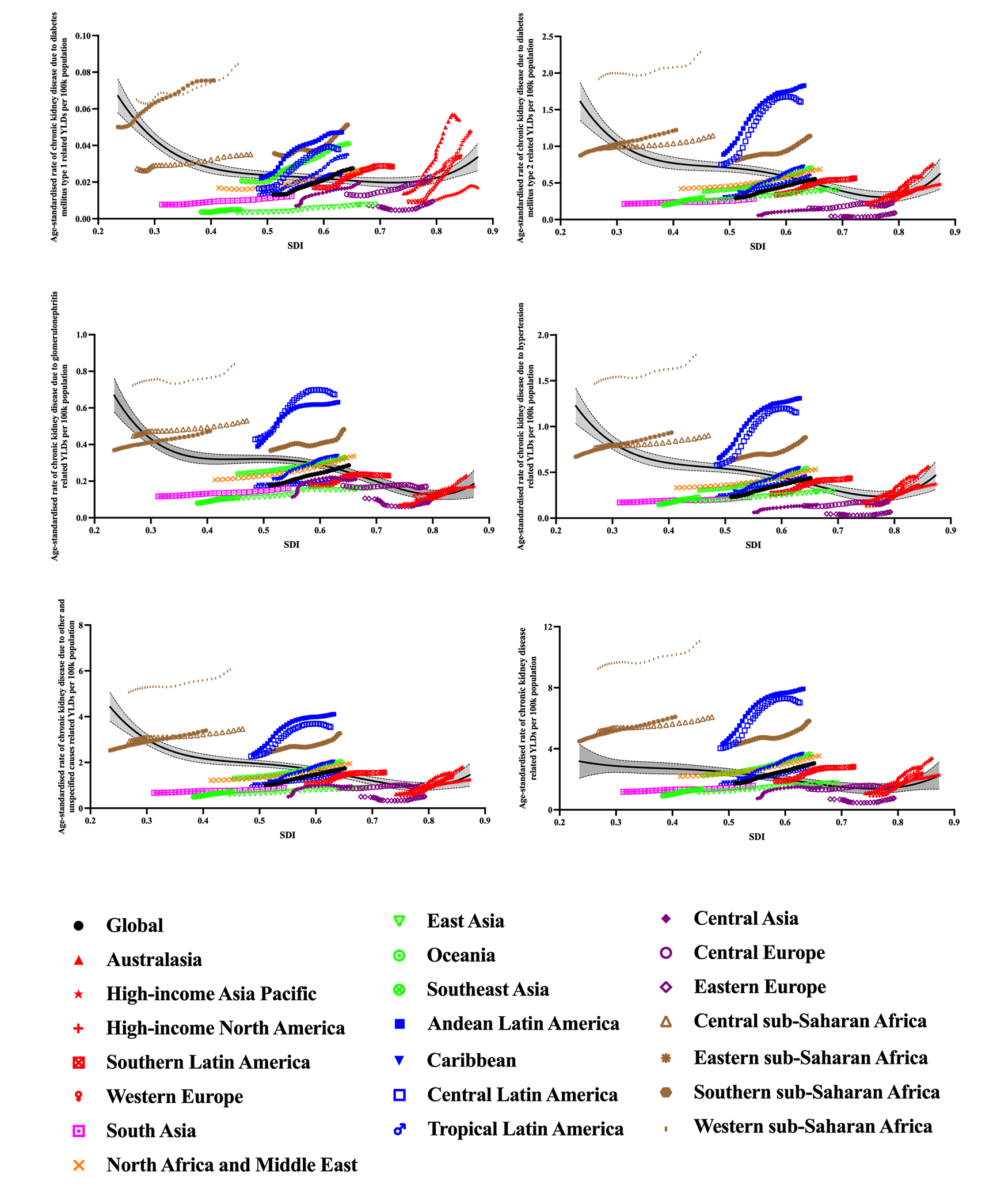

Supplement: SUPPLEMENTARY FIGURE 2 — Trends in YLDs of heart failure caused by chronic kidney disease for 21 GBD regions by SDI, 1990–2021. For each region, points from left to right depict estimates from each year from 1990 to 2021. ASR, age-standardized rate; SDI, Socio-demographic Index; YLDs, Years lived with disability. [file Image_2.tif]
