# Supplementary material for: Global trends and disparities in the burden of heart failure caused by chronic kidney disease: an analysis of the global burden of disease study 2021
Source: Front Med (Lausanne). 2025 Apr 17;12:1567128. doi: 10.3389/fmed.2025.1567128 (PMC12043581; doi:10.3389/fmed.2025.1567128)
Supplement: Supplementary file 3 [file Data_Sheet_1.docx]

**Additional file 3**

**Prediction Model Method**

Auto-regressive Integrated Moving Average model (ARIMA) was applied in the prediction process. It is specified by three main components, P, D and Q. P stands for auto-regression, representing the number of lag observations in the model; D stands for integration, representing the number of times that input raw data are differenced, making the model stationary; Q stands for moving average, representing the size of moving average window applied to lagged observations. For non-stationary data, to eliminate the non-stationarity, differencing step will be applied once or more. The model was established by Python. First, analyze the data and compute the difference between consecutive data to choose the P, which makes the data stationary. Then, loop the steps “p<data.size/10,q<data.size” to generate all combination of P and Q, and choose the parameter with the minimum Bayesian Information Criterion. Finally, generate prediction. The code is as follows:

#coding=gbk

import matplotlib.pyplot as plt

import pandas as pd

from statsmodels.tsa.arima_model import ARIMA

from statsmodels.graphics.tsaplots import plot_acf, plot_pacf

filename = r'death+rate+data .csv'

data = pd.read_csv(filename,usecols=['Year','Male'] ,index_col = u'Year',dtype={'Male': float})

plt.rcParams['font.sans-serif'] = ['SimHei']

plt.rcParams['axes.unicode_minus'] = False

init_d= 1

D_data = data.diff(init_d).dropna()

D_data.columns = [u'Male']

pmax = int(len(D_data) / 10)

qmax = int(len(D_data) / 10)

bic_matrix = []

for p in range(pmax +1):

temp= []

for q in range(qmax+1):

try:

temp.append(ARIMA(data, (p, init_d, q)).fit().bic)

except Exception:

temp.append(None)

bic_matrix.append(temp)

bic_matrix = pd.DataFrame(bic_matrix)

p,q = bic_matrix.stack().idxmin()

model = ARIMA(data, (p,init_d,q)).fit()

model.summary2()

forecast, fcasterr, conf_int = model.forecast(20)

for x in forecast:

print
